# Editorial

**DOI:** 10.1590/S1980-57642008DN10300001

**Published:** 2007

## Ethical aspects of end-of-life

In this issue of **Dementia & Neuropsychologia**, the News and Views section includes an article on the physician’s attitude and conduct when a patient reaches the terminal phase of a severe and incurable illness. This situation, which not rarely challenges physicians caring for patients with dementia or other chronic diseases, is comprehensively analyzed in the article.^1^ A recent Brazilian Federal Medical Council resolution states that: “Doctors are permitted to limit or suspend procedures and treatment which prolong life of patients in a terminal phase of a severe and incurable illness, respecting the person’s will or that of their legal representative”. Although this is very clear statement, Oselka and Oliveira also included opinions of religious leaders and viewpoints of respected Brazilian attorneys and jurists on end-of-life, to present a deep and objective analysis of this complex matter.^1^

## Informant questionnaire versus real performance

Another important aspect for clinical and even basic research is related to the translation of scales originally designed and used in other countries with different languages and cultures. This subject raises several controversial questions regarding the methods of translation and adaptation, the need for validation, the number of participants included in such validation studies, and so forth. In this issue, Bressan and colleagues^2^ bring up another interesting issue regarding the use of an informant-based questionnaire of functional activities (FAQ^3^). The results of the FAQ were compared with those based on observations by the patients actually performing these activities in the questionnaire. The authors found significant differences amongst the results, showing that in their sample the informants tended to underestimate patients’ competency. Although these findings should be interpreted with caution and need replication by other researchers, they are important because the FAQ has been used by several epidemiologic studies in Latin America.^4-7^ Commencing in 1989, a World Health Organization cross-national project proposed the combined use of a version of the FAQ and the Mini-Mental State Examination as a screening tool for dementia.^5,8,9^ Chile was included as one of the six countries participating in this project, and the Chilean version of the FAQ^5^ was translated into Portuguese and adapted for use in Brazil. It should be noted that the version used by Bressan and colleagues was directly translated from the original FAQ, while the cited Brazilian epidemiologic studies^4,6,7^ have used the adapted version from that translated in Chile.

## First Brazilian Symposium of Frontotemporal Lobar Degeneration (FTLD)

Frontotemporal dementia, primary progressive aphasia and semantic dementia are the main conditions included in this group of brain degenerative diseases. Initially considered rare disorders, and also included in the group entitled “Non-Alzheimer dementia”, FTLD has come to the fore over the last few years, after new and relevant clinical and molecular biology findings have brought it center stage in the dementia research field. As there are increasing numbers of Brazilian studies in FTLD, it was felt appropriate to initiate regular meetings where researchers may liaise with each other and with renowned colleagues from abroad. It is a privilege for **Dementia & Neuropsychologia** to be able to publish the abstracts of the first Symposium in this issue.
